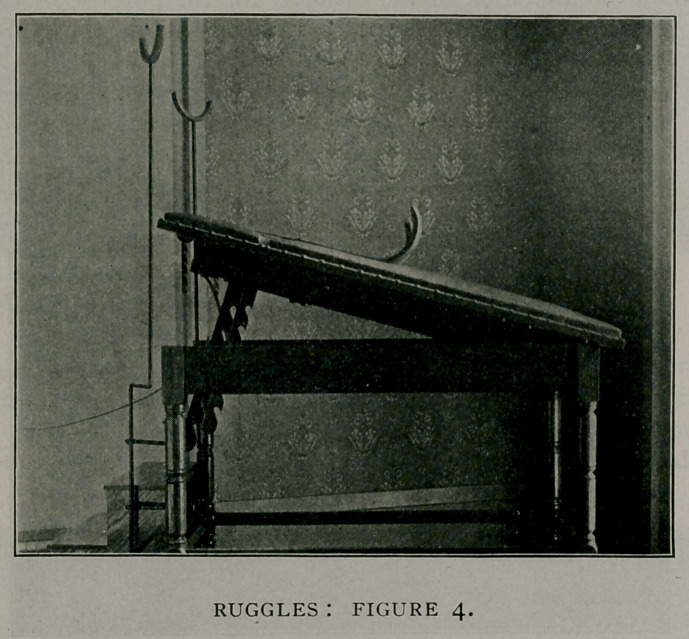# A New Genitourinary Table1Demonstrated at the annual meeting of the Medical Association of Central New York, held at Rochester, October 18, 1904.

**Published:** 1905-03

**Authors:** E. Wood Ruggles

**Affiliations:** Rochester, N. Y.


### A New Genitourinary Table.[1]

By E. WOOD RUGGLES, A. M., M. D., Rochester, N. Y.

I WOULD not take up the time of this association, the members of which are most interested in general subjects, with the demonstration of this table which I have devised for my own particular line of work, if it were not equally applicable to general purposes. Indeed, I never thought of showing it at all, until a general practitioner who examined it was so pleased with its working that he requested me to give him its measurements and directions for its manufacture, so that he might have one made for himself. It certainly answers my own purposes very much better than any table I have ever seen, not excepting the elaborate and expensive genitourinary tables designed by Drs. Tilden Brown, of New York, and Bransford Lewis, of St. Louis.

I will first show it as a regular six-foot table (Fig. 1) for massage, the passage of sounds, palpation of kidneys, examination of varicocele, hernia and all conditions where a recumbent posture is desirable. For these purposes its height (36 inches), which saves tiring the physician's back is its chief recommendation.

For gynecological examination and treatment, one of the following positions will recommend itself to every one: first, with the table level, the feet being elevated to the same height by the foot-rests (Fig. 2); second, with the pelvis elevated either slightly or sharply and the back elevated also, according to desire (Fig. 3). As will be seen in Fig. 2, a douche-pan is at-

1. Demonstrated at the annual meeting of the Medical Association of Central New York, held at Rochester, October 18, 1904.

RUGGLES: FIGURE I.

tached to the table, which can be drawn out when it is desirable to irrigate the vagina, bladder or male urethra. The end of the table, over this douche-pan, has a segment of a circle, 10 inches long and 2½ inches deep, cut out at the center, thus making

RUGGLES: FIGURE 2.

irrigation and the use of instruments much more convenient. A corresponding piece on the detachable end section of the table fills this gap, when using it as a full length table.

For urethroscopic examination, either of the anterior or posterior urethra, I prefer the position in Fig. 3.

For cystoscopy, with water dilation, the same position of the pelvic section, but with the back only slightly elevated, is preferable and is that used by Nitze, of Berlin, the originator of cystoscopy.

With air dilatation of the bladder, for the purpose of examination of the bladder walls or for catheterisation of the ureters,

RUGGLES: FIGURE 3.

it is necessary to tilt the pelvis well upward, in order to divert the residuum of urine or the cleansing solution used, which always remains in the bladder, from the urethral orifice. This is accomplished by most operators by putting the patient in the Trendelenburg position (Fig. 4.) The straps holding the supports for the shoulders are hung from the center of the end and diverge, because, if in a parallel position, they tend to let the patient slide down between them and away from the operator, owing to the sloping of the shoulders. The leg rests support the knees in a pretty comfortable position. This position can also be used for any operation requiring the Trendelenburg position and can be varied to suit the operator.

For cystoscopy, however, this position is very uncomfortable for the patient, if not anesthetised, and is avoidable by very slightly elevating the back, and raising the pelvic section to the top notch, a modification of the position in Fig. 3. Air dilatation is quite distressing enough to the patient without the added discomfort of running most of his blood into his head and piling his abdominal contents on top of his heart and lungs.

A representative of one of the cystoscope companies, who has performed over 2,000 cystoscopies said that this table was the

RUGGLES: FIGURE 4.

only one which gives the position he has found best for cystoscopy. He generally uses a level table, with a bag of sand at the extreme end to elevate the pelvis, but he says that the slight elevation of the back with this table restrains the patient from slipping off and away from the operator. Air dilatation has been tremendously popular in America, but there is at present a decided reaction in favor of water dilatation, as far less painful and more practical, except for local treatment and operation on the bladder walls.

294 ALEXANDER STREET.

---

A NEW polyclinic for the relief of the sick poor is about to be established at Athens in the Piræus. It is to be entirely managed and officered by women.

## Figures and Tables

**Figure I. f1:**
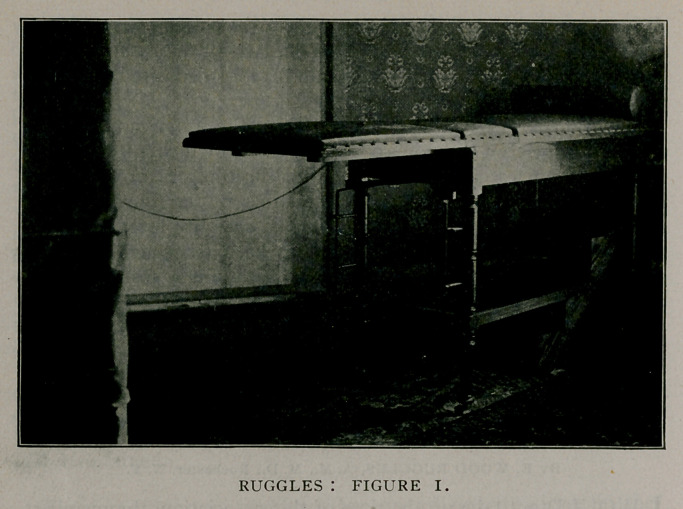


**Figure 2. f2:**
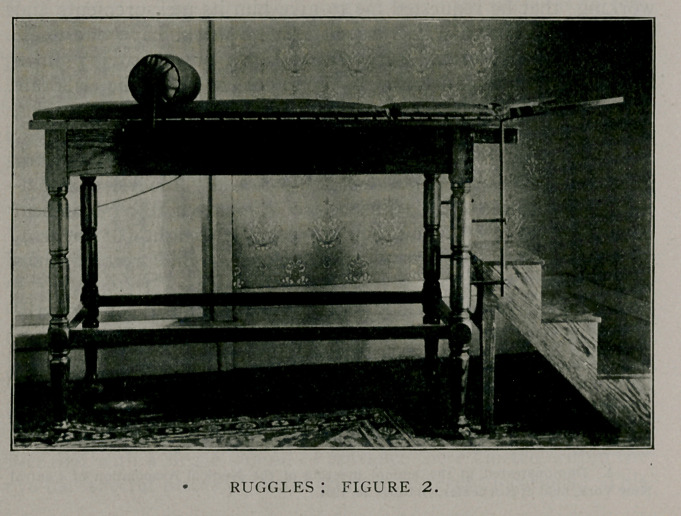


**Figure 3. f3:**
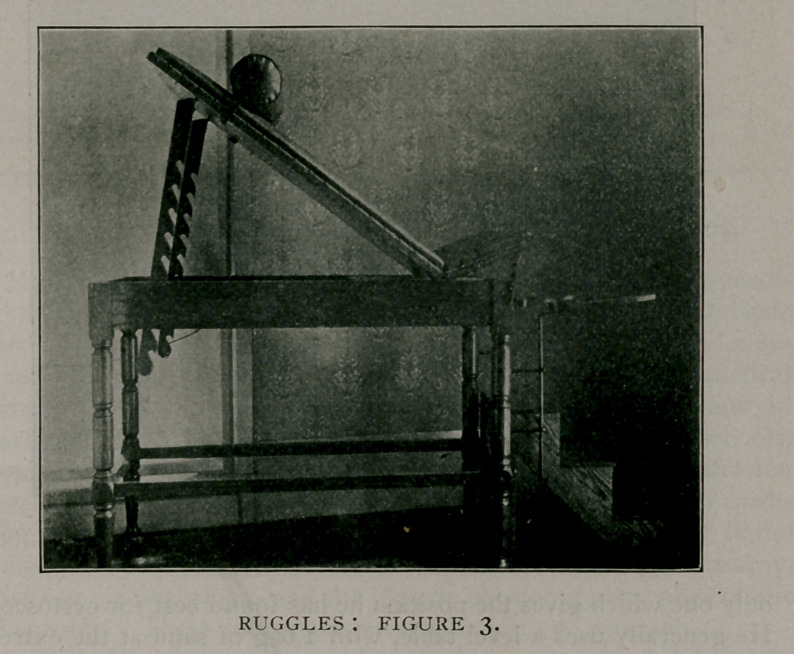


**Figure 4. f4:**